# Topical L-Ascorbic Acid Formulation for a Better Management of Non-Melanoma Skin Cancer: Perspective for Treatment Strategies

**DOI:** 10.3390/pharmaceutics13081201

**Published:** 2021-08-04

**Authors:** Pier Cesare Capponi, Domenico Murri, Carmine Pernice

**Affiliations:** 1 Radiopharmaceutical Chemistry Section, Azienda USL-IRCCS di Reggio Emilia, 42123 Reggio Emilia, Italy; 2 Otolaryngology Unit, Department of Surgery, Azienda USL-IRCCS di Reggio Emilia, 42123 Reggio Emilia, Italy; Domenico.Murri@ausl.re.it (D.M.); Carmine.Pernice@ausl.re.it (C.P.)

**Keywords:** non-melanoma skin cancer (NMSC), basal cell carcinoma (BCC), squamous cell carcinoma (SCC), topical formulation, skin cancer, L-ascorbic acid (AA), ascorbyl radical (Asc •), hydrogen peroxide (H_2_O_2_), biodistribution, pharmacokinetics

## Abstract

L-ascorbic acid, is a well-known molecule, sometimes used as antioxidant for skin care. Nonetheless, few studies have taken in account its utility as topical treatment for non-melanoma skin vancer. Non-melanoma skin cancer includes basal cell carcinoma and squamous cell carcinoma and is widespread worldwide with an increasing incidence. The purpose of this paper is to analyze the characteristics of L-ascorbic acid topical formulation, its percutaneous absorption and biochemical mechanism, focusing on its anti-cancer properties. In particular, it will be described how the pH and the concentration of the formulation are able to influence its distribution in the skin and tissues. We will report, the current knowledge on the pharmacokinetic aspects of L-ascorbic acid that allows us to reconsider it in the light of its ability to act as a prodrug and as an anticancer agent. Lastly, a short review with the aim to find any evidence of a possible clinical use of L-ascorbic acid for the treatment of non-melanoma skin cancer was made.

## 1. Introduction

The use of L-ascorbic acid (AA) to treat cancer is not a new concept, in the 1970s, Cameron, Campbell, and Pauling [1,2,3], reported that high-dose intravenous of this antioxidant, increased the survival rate of advanced cancer patients. Since then, much research has been conducted to improve understanding of the biological activities of AA, which has led to a number of plausible mechanisms for its anti-cancer activity. A growing number of studies have demonstrated that mM concentrations of pharmacological AA, can kill cancer cells in vitro and slow tumor growth in vivo. However, the exact mechanism by which some cancer cells are sensitive to AA, while normal cells remain resistant, is still poorly understood. The main studies of the last 50 years relating to the antitumor activity of AA are mainly based on oral or parenteral administration and therefore, on a systemic use of this molecule. Recently, some research has shown that the efficacy of AA as an anticancer agent, is linked to the achievement of pharmacological concentrations of the molecule near the tumor cells. From these studies it has emerged that the pharmacokinetics of AA would play a leading role [4,5,6,7].

In this review, we show how some clinical evidence has shifted the interest for topical use of this molecule in non-melanoma skin cancer (NMSC).

Although surgery and radiotherapy remain the gold standard treatment for NMSC, new topical treatments could be used with the aim to reduce or avoid the incidence and the extent of surgical excision. The use of AA topical formulations brings to light new treatment perspectives that could lead to an improvement in current clinical practice. Topical use of AA has already been used in the dermatological field [8], as an antioxidant for skin care but only, recent clinical cases, has shown its effectiveness in the treatment of BCC and SCC [9,10,11], two skin cancers extremely common in the population. These studies describe the use of a formulation with adequate chemical-physical characteristics, able to guarantee the passage through the stratum corneum and to determine its therapeutic efficacy. Despite numerous studies have investigated the functions of AA as a structural physiological component of the skin, its role as an anticancer agent in skin cancers is still poorly understood.

Here, we describe the properties of the AA-based formulation, focusing on the rationale and strategy for its translational clinical use for skin cancer.

## 2. L-Ascorbic Acid as a Pro-Drug

Over the years, AA has been widely debated for its anticancer properties and numerous studies have tried to determine its effective efficacy and its mechanism of action. Among the many hypotheses formulated on the anticancer mechanism of AA, some of these present an explanation on the variability of the clinical data relating to its pharmacokinetics for systemic use [5,6,7]. In this context, experimental data conclude that the mechanism by which AA determines toxicity towards tumor cells is greater in extracellular fluids than in blood and is closely linked to its bioavailability. This concept offers a new interpretation for a potential new clinical use of AA in the dermatology and oncology field. The effectiveness of AA, has often been tested using different routes of administration, often neglecting the aspects related to its biodistribution in tissues and organs. Furthermore, the complex pharmacokinetics of AA, have often been overlooked in the design of intervention studies, resulting in erroneous interpretations and conclusions. Regarding this, it has been recognized that the route of administration could produce large differences in plasma concentrations [5,6,7]. Steady-state plasma AA values were observed, with up to 70-fold differences in plasma concentrations, between oral and i.v. administrations [6]. In oral administration, the control of intracellular and extracellular AA concentrations is subject to three mechanisms: intestinal absorption, tissue transport and renal reabsorption and excretion [6]. These three mechanisms work in a coordinated way, ensuring that the AA is tightly controlled. Conversely, parenteral administration bypasses these controls, which are restored when the kidneys excrete AA, when concentrations are higher than those corresponding to the Vmax of the resorption transporters. Parenteral administration produces a predictable plasma concentration, avoiding absorption limits, resulting in 100% bioavailability. However, the distribution of AA depends on the vascularity of the various tissues and plasma concentrations do not appear to influence the normal distribution of the tissue beyond saturation [4]. Other factors could play a role in the absorption and distribution of AA in the body and the mechanism behind the highly differential steady-state concentration of AA in various tissues remains largely unknown. AA transporters with specific tissue expression were identified [4,5,8] and the possible role of passive distribution in the absorption and biodistribution of AA was also highlighted. AA distribution between organs and tissues across biological membranes can vary due to the ox-redox state of the molecule (AscH^−^, DHA), the pH of the environment and the concentration gradient of the AA between the compartments. All of these factors contribute to the regulation of AA pharmacokinetics, which shows a compartmentalized distribution pattern with a wide range of organ concentrations of AA. It is assumed that these differences in concentration in plasma and in the various body compartments are related to a series of control systems with kinetics not yet fully elucidated. Furthermore, the absorption and distribution of AA can be influenced by several factors, including genetic and environmental polymorphisms, lifestyle factors such as smoking and diet, as well as diseases. In this context, it should be noted that many cancers can be poorly supplied with blood or have an altered vascularization resulting in less accumulation of AA near the tumor cell. The absorption, distribution, metabolism, and excretion of AA in humans is very complex, still poorly understood and different from that of most low molecular weight compounds.

Given the new interests regarding the pharmacokinetic profile of AA, the attention of researchers and clinicians has also focused on the pharmacodynamic aspects of the molecule.

A mechanism for AA anticancer activity has been proposed, according to which the molecule behaves as a pro-drug for the selective transport of hydrogen peroxide (H_2_O_2_) into the extravascular space near cancer cells [6,7].

Therefore, the peroxide (H_2_O_2_) would be the effective drug, capable of causing toxicity towards cancer cells through a series of selective cytotoxic mechanisms that involve in depletion of ATP leading to cancer cell death [12,13]. AA only at specific pharmacological concentrations (0.3–20 mM), versus physiologic (0.1 mM), is able to determine toxicity toward tumor cells, through the formation of H_2_O_2_, starting from the radical of AA (ascorbyl radical (Asc •)) [5,6,7]. Based on in vitro and in vivo experimental tests, Levine et al. [5] and Qi Chen et al. [5,6] proposed a mechanism for the generation of H_2_O_2,_ starting from ascorbate mono anion (AscH^−^).

The presence of AA in equilibrium with its anionic form (AscH^−^), can determine the reduction of the endogenous iron contained in the metal proteins, where Fe^3+^ is reduced to Fe^2+^ with the formation of Asc • (**REACTION 1**). Subsequently the Fe^2+^ donates an electron to the molecular oxygen, with the formation of active oxygen including superoxide (O_2_^• −^) (**REACTION 2**) with subsequent dismutation in H_2_O_2_ (**REACTION 3**).

The reaction complex follows a multi-step process:

**Reaction 1. (Formation of Asc •)**

AscH^−^ + Fe^3+^ → Fe^2+^ + Asc • + H^+^

**Reaction 2. (Formation of active oxygen)**

Fe^2+^ + 2O_2_ → Fe^3+^ + 2O_2_ •

**Reaction 3. (Dismutation in H_2_O_2)_**

2O_2_ • + 2H^+^ → O_2_ + H_2_O_2_

Therefore, the formation of Asc • is closely linked to the presence of metals, hypothetically identified within proteins. The reactivity of AA towards metals has been known for a long time [14], indeed AA is easily oxidized by Fe^3+^ ions through an intermediate chelate complex, forming a stable Asc •.

On the other hand, it is also known that the redox-active Fe^2+^ ion in the presence of peroxides, initiates a cascade of redox reactions with the formation of ROS (Fenton Reaction) [14]. However, only recent research has hypothesized an in vivo mechanism, in which the electron lost from ascorbate ion (mono anion) selectively induces H_2_O_2_ formation in extracellular fluids. The peroxide generation process is mainly tissue-specific, as in vivo tests have shown that Asc • and H_2_O_2_ are mainly detectable in the extracellular fluid but not in the blood [6,7]. A series of in vivo experimental tests were conducted to explain the preferential Asc • and H_2_O_2_ formation in extracellular fluid over blood, after parenteral administration of AA. The results obtained supported the hypothesis that in the blood, the ability to form H_2_O_2_, starting from Asc •, is very reduced compared to extracellular fluids, because its formation is inhibited by the presence of reducing proteins, not equally distributed in the extracellular space [4,5,6,7].

Interestingly, amounts of H_2_O_2_ are normally produced in metabolic, physiological and pathological processes such as in macrophage phagocytosis. Therefore, biological systems are equipped with enzymes to preserve cellular integrity from the action of peroxides and oxidizing species.

Red blood cells normally contain large amounts of enzymes capable of preserving hemoglobin from oxidative damage and capable of neutralizing the circulating H_2_O_2_ in the blood.

Forman et al. [15] described that the H_2_O_2_ generated in the blood, can enter in red blood cells, through aquaporins, the protein channels generally associated with water transport and the intracellular removal of H_2_O_2_ is catalyzed by enzymes like catalase and glutathione peroxidases.

The main physiological mechanisms of peroxide elimination are described below:

Catalase dismutates H_2_O_2_ to O_2_ and H_2_O:

2H_2_O_2_ → O_2_ + 2H_2_O

The glutathione peroxidases catalyze the reduction of H_2_O_2_ by glutathione:

H_2_O_2_ + 2GSH → GSSG + 2H_2_O

The combined action of these enzymes contributes to an efficient removal of peroxide from the blood and this does not allow to reach systemic doses of H_2_O_2_ that could be effective against cancer cells.

To understand the pharmacology of AA, the next key point is related to the formation of Asc •, also present mainly in extracellular tissues.

Qi Chen et al. [6], showed that Asc • concentrations in the extracellular fluids of mice and rats were detectable over a wide range of administered doses of AA, while in blood it was not evident.

The data suggest that the half-life of Asc • in blood is also limited and that the process required, to form peroxide, is related to the extravascular compartment.

AA is a hydrophilic molecule subject to oxidation which in oral or parenteral administration, is subject to rapid elimination by the body. The limitations due to the achievement of active Asc • and H_2_O_2_ concentrations in extracellular tissues, are also related to the pharmacology of the parent molecule AA [5].

AA is subject to a series of physiological control mechanisms that limit its bioavailability in oral administration, such as intestinal saturation. Alternatively, the parenteral administration, although capable of reaching plasma concentrations higher than those possible with the maximum tolerated oral doses, does not seem able to guarantee a constant efflux into the extracellular fluids. It has been estimated that Asc •, in extracellular fluids, must have a concentration >100 nmol/L to generate H_2_O_2_ [6,7] and it is important to note that the presence of Asc • shows a linear relationship with the initial amount of AA present in the blood.

Some authors have pointed out [4] that there is a significant difference in the accumulation capacity of AA in tissues when plasma levels are in the ‘physiological’ (50 mM) or supra physiological (100 mM) range. However, the supra physiological levels of AA required to overcome this diffusion limit from plasma to extracellular tissue are not easily achievable.

A further observation is related to the exposure of the pharmacological concentrations of the Asc • which must be constant and lasting over time, to develop toxicity in tumor tissues [6].

In this context it can be assumed that the selective toxicity of peroxides requires high concentrations and sufficiently long times to kill cancer cells.

A further point of interest is linked to the selective toxicity towards cancer cells by H_2_O_2_.

It should be noted that the concentrations of H_2_O_2_ induced by pharmacologic AA are far higher, as much as two orders of magnitude, than those concentrations that regulate normal cellular processes, however these high concentrations do not affect healthy cells.

As proposed by Doskey et al. [12], this toxicity would selectively affect cancer cells, sparing healthy cells, due to the lack of enzymes resistant to H_2_O_2_.

It was observed that normal cells have a more robust ability to remove extracellular H_2_O_2_ than cancer cells. In the latter, the catalase activity revealed a lower ability to remove peroxide. Levine et al. [5,6,7] hypothesize that normal cells have redundant mechanisms for H_2_O_2_ disposal and/or repair of H_2_O_2_ damage. In contrast, susceptible cancer cells may have a series of mutations that signal cell death in the context of H_2_O_2_ formed by pharmacologic AA.

Pharmacokinetic data, are essential for recognizing a clinical translation potential and the key to advancing knowledge, is that H_2_O_2_ is formed only with pharmacological but not physiological AA concentrations. This last concept provides a solid rationale as a prerequisite for re-examining AA in cancer in the light of pharmacokinetic aspects.

In conclusion, the key points for a selective toxicity towards cancer cells are the generation of significant quantities of H_2_O_2_ (mediated by metal-protein catalyst) and by supra physiological concentrations of AA, which must be constant and continuous over time to generate the death of cancer cells.

## 3. Chemistry of the L-Ascorbic Acid and Its Free Radical

**Figure 1:**
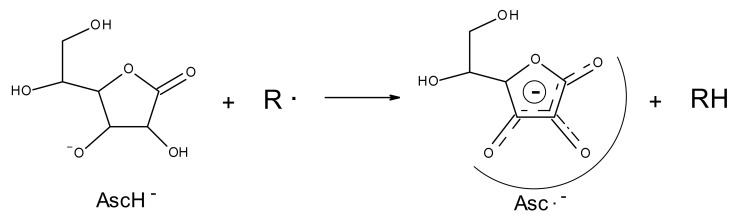
Conversion of the mono anion form of AA in Asc • by the transfer of an electron. The stability of the Asc • is due by the unpaired electron delocalized in the tricarboxylic ring.

**Figure 2:**
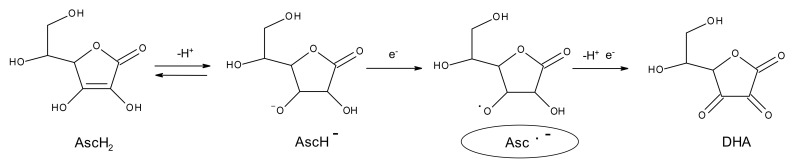
Ox-redox states of ascorbic acid.

AA is usually known to be an antioxidant with reducing capacity, due to the presence of an enediol group, however in a particular ox-redox state its action can be different, in fact, in presence of oxygen, AA tends to oxidize and form dehydroascorbic acid (DHA). AA can be oxidized by losing two protons and two electrons, but normally loses only one electron at a time, to form, in the first step mono anionic ascorbate (ionic form) and in the second step dehydroascorbic acid (DHA). When AA is oxidized, the two-step oxidation involving a free radical intermediate, known as ascorbyl radical (Asc •) (Figure 1 and Figure 2). Asc • is a transient species that derives from the conversion of the ascorbate ion (prevalent form at physiological pH) by the transfer of an electron (Figure 1). The reactivity of the Asc • is singular, in that it can either disproportionate or react with other radicals, but it reacts poorly with non-radical species. In the 1970s, Bielski et al. [16] observed that AA is a reducing agent and his radical is an oxidant, evauating its stability. The Asc • is stable, for being a radical species, because the unpaired electron is delocalized in the tricarboxylic ring (Figure 1) and is stable enough to be metabolized and excreted from the body as such. Although this transient species has since been known, only little information on its interaction chemistry with other compounds of biological importance has been studied. Studies have shown that the presence of this transient species is a necessary intermediate for the action of AA as an anticancer agent. Therefore, it is of interest to understand not only the reactivity of AA toward biological compounds but also the reactivity of his radical.

## 4. L-Ascorbic Acid in the Body

AA exists mainly in two forms in vivo, the reduced form AscH_2_ and DHA (oxidized form), of which the first is by far predominant in the ionic form AscH^−^ (at physiological pH). Most cell types are able of effectively reducing DHA to AscH^−^ and thus the total capacity of AA available in the whole organism is considered to be the combined pool of AscH^−^ and DHA [17]. The turnover of AA is therefore particularly linked to the catabolism of DHA which occurs by hydrolysis to 2,3-Diketogulonic acid and decarboxylation to l-Xylonate and l-Lyxonate, both of which can enter the pentose phosphate pathway for further degradation [17]. The metabolism of AA does not involve conjugation reactions: in contrast to plants, where a number derivatives and analogues, including several glycosides have been identified, only non-conjugated forms of AA are present in mammals [17]. Ingested AA is absorbed through the intestinal epithelium, mainly by sodium-dependent VitC Transporters (SVCT) and reaches the blood where a part is taken from the erythrocytes. The latter are able to recycle DHA into AscH^−^ and maintain an intracellular concentration of AA similar to that of plasma. The recycling capacity of erythrocytes can constitute a consistent antioxidant reserve in vivo for both erythrocytes and for the whole organism. Intracellular concentrations of AA range from approximately 0.5 to 10 mM compared to only 50–80 µM in the plasma of healthy individuals, confirming a multiple preference for tissues. AA is 90–95% present in plasma in the form of AscH^−^ and 5–10% as DHA [17]. A part of the circulating pool is stored in the body’s tissues, particularly in the adrenal glands and liver while the part not stored is eliminated in a short time (2–4 h). AA is a highly hydrophilic low molecular weight compound and it is effectively excreted via the kidneys. The AA is quantitatively filtered through the glomerulus by the hydrostatic pressure gradient and concentrated in the pre-urine following reabsorption of water [17].

## 5. Specific Tissue Activation and AA Distribution

Pro-drugs are inactive precursors that are bio-transformed into active metabolites by the action of enzymes or by biochemical mechanisms. Prodrugs can be intentionally designed to overcome pharmacokinetic problems, for example to give the drug site-specific activation in organs or tissues.

To consider AA as a pro-drug, it is important to analyze its site-specific activation in extracellular tissue mediated by metal proteases.

Levine et al. [5] hypothesized that in the interstitial space of the tumor, AA is led to form its radical (Asc •) starting its mono anionic form (AscH^−^). This reaction (**Reaction 1**) occurs mainly in extracellular tissues where suitable metal catalysts are present, while in the blood, these reactions are reduced to a minimum.

The identity of a specific metal catalyst required for the action of pharmacological AA is currently unknown and although Asc • formation occurs even to a lesser extent in the blood, experimental data [5,6,7] suggest that metal proteases may be present with high affinity for AA in the extracellular space of tissues and tumors. In control animals, pharmacological concentrations of AA added exogenously to the formation of Asc • and H_2_O_2_ induced by the collected extracellular fluid, indicating that the components of the extracellular fluid were required for the reactions [6]. Although not identified, some hypotheses have been made about the nature of the catalytic species. Free metals are normally present in extracellular tissues, however free iron and copper are very unlikely to be mediators, as these cations are closely linked in vivo. The authors suggest that may be more candidate metal–protein catalysts that would have a high Km for AA and/or molecular oxygen, to become permissive towards this process, only when AA is present in sufficient pharmacological concentrations.

All the hypotheses discussed above require adequate access for AA to tumors cells with effective distribution throughout the extracellular environment near the tumor.

Therefore, the critical point is related to the distribution of AA from the blood to the tissues.

In a recent review [4], the distribution of AA through extracellular tissue and into tissue cells was described in relation to plasma concentration. It has been reported that when plasma levels are low (10 mM), AA is unable to reach the tissues. These plasma levels represent a state close to scurvy (complete deficiency). It was observed that there is a significant difference in the capacity for AA to accumulate in tissue cells when plasma levels are in the ‘healthy but not saturated’ range (50 mM) or saturated (100 mM).

Below saturation, only cells adjacent to the blood vessel wall accumulate physiological intracellular concentrations and the diffusion zone within the tissues does not extend beyond certain thresholds, even with high plasma AA concentrations [4].

Cells distant from vessel walls in deep tissues cannot accumulate AA, so supraphysiological levels are required to overcome this diffusion limit. These data show that AA is unable to diffuse effectively deep from the blood to extracellular tissue and oral and parenteral administration is unlikely to ensure effective concentrations near deep tumor masses.

In conclusion, metal protease-mediated site-specific activation occurs predominantly in extracellular tissues and the distribution of AA from blood to extracellular tissues is limited. One form of administration that may allow AA to reach effective concentrations in the extracellular space by bypassing the blood is topical administration through the skin, as we will see in the cases considered in this review.

## 6. Physiological Role of AA in the Skin

The skin in humans and mammals is the largest, most dynamic and regenerating organ and comprises about 15% of the total body weight. It has the function of protecting the underlying tissues (muscles, bones, internal organs) from external insults.

The skin performs several important functions for the homeostasis of the organism and one of its main roles is represented by its function as an anatomical barrier against potential pathogens.

Another function of this organ is represented by sensitivity, in fact it consists of numerous nerve endings that detect changes in temperature and pressure and mediate the sense of touch and sensitivity in general.

Furthermore, it controls evaporation by forming a dry and relatively impermeable barrier against the loss of liquids, regulating the excretion of electrolytes and waste substances through sweating.

The skin acts as a thermoregulator of body temperature, modulating the activity of blood vessels and preventing the loss of water and it plays a role of synthesis and reserve of lipids and water necessary for the correct maintenance of its functions.

To maintain its elastic, structural, and functional properties, the skin needs numerous substances, including AA. Inside, AA supports important and well-known functions, such as: stimulating collagen synthesis and assisting antioxidant protection against UV-induced photodamages. The skin, therefore, is characterized by physiological concentrations of AA present both in the dermis and in the epidermis [8]. Most of the AA contained in the skin appears to be contained in the intracellular compartments in a concentration of mM order [8].

**Figure 3:**
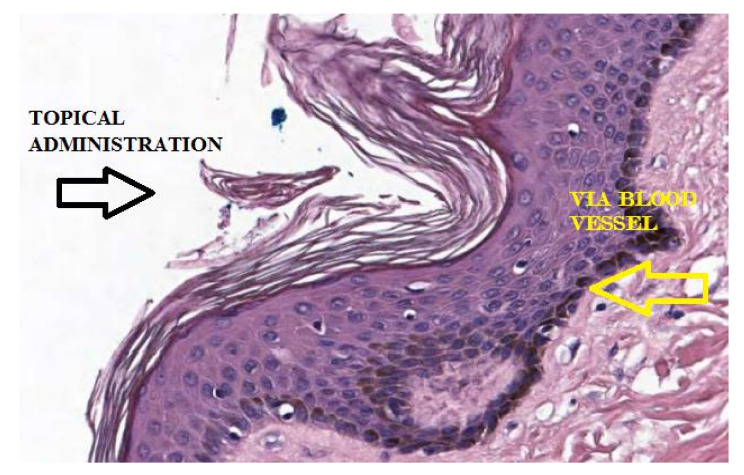
Enlarged representation of the skin. In pink, the layer of the dermis, supplied by blood vessels that allow the transport of nutrients towards the epidermis in dark purple.

The physiological distribution of AA in the skin from the plasma occurs via a flow of nutrients from the blood vessels in the dermis to the epidermal layer (Figure 3).

Transport through the skin layers is mediated by specific sodium-dependent vitamin C transporters (SVCTs) which are present throughout the body and are also responsible for transport to other tissues [4,5,8,17].

AA actively accumulates in epidermal and dermal cells via the two sodium-dependent AA transporter (SVCT) isoforms 1 and 2 [8].

The presence of this specific transporter suggests that AA has crucial functions within skin cells. Both transporters are hydrophobic membrane proteins that co-transport sodium, driving the uptake of AA into cells and exhibit different absorption kinetics reflecting their different physiological functions.

Interestingly, epidermal cells express both types of transporters. Most tissues mainly express SVCT2, while SVCT1 expression in the body is largely limited to cellular epithelium in the small intestine and kidneys [8,17]. The presence of both transporters suggests that AA plays an important role in this organ, possibly maintaining adequate concentrations; however, it is not known whether the transporters have an implication in percutaneous distribution and absorption of AA. It has also been observed that AA is found more in the epidermal layer than in the dermis, with differences of 2–5 times between the two layers. Furthermore, there appears to be a concentration gradient between the stratum corneum and the epidermal layer [8]. AA at lower concentrations would be present on the outer surface of the epidermis and higher concentrations in the deeper layers of the stratum corneum. This could reflect the exhaustion of external cells due to chronic exposure to the external environment.

## 7. Passive Diffusion of AA and Implications for Its Topical Use

**Figure 4:**
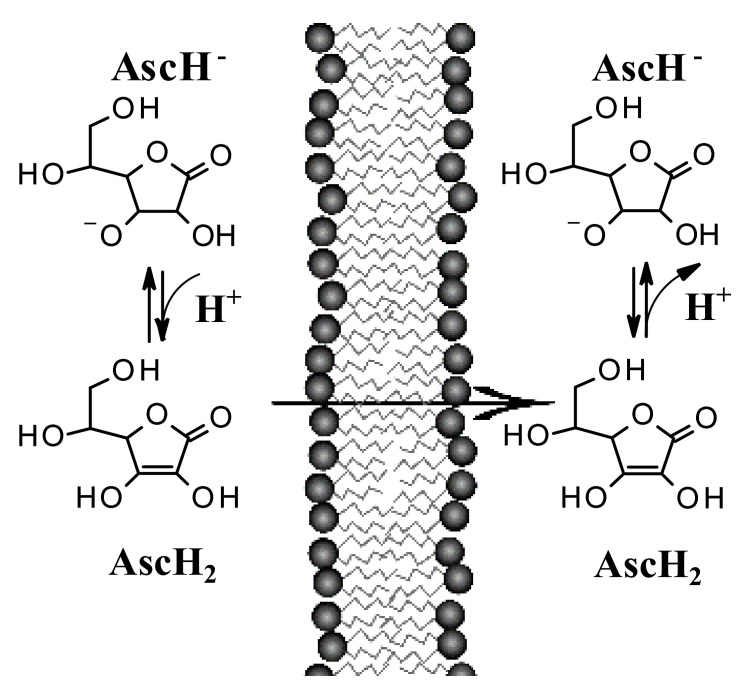
Only the protonated form AscH_2_ is able to cross biological membranes by passive diffusion. The monoanionic form is unable to pass lipid membranes.

While the processes of distribution, metabolism, and excretion are well known for systemic drugs, the steps of percutaneous absorption of topical drugs or molecules are not always well known. The skin permeability of a drug is severely limited by the stratum corneum, due to the low permeability of its cellular elements. Absorption through this layer can occur lipophilically (transcellular), hydrophilically (intercellular), and through the hair follicles. The contribution of the latter way is generally of little importance, while most of the molecules permeate the stratum corneum using both intracellular and extracellular ways. Efforts have been made to develop derivatives of AA to increase its penetration into the skin and to ensure the stabilization of the molecule from oxidation [8]. However, in the context of its use, as an anticancer, it is not known whether changes in the molecular structure could negatively affect its mechanism of action. The addition of a phosphate group gives greater stability and these derivatives can be converted to AA in vivo, but are poorly absorbed through the skin. Ascorbyl glucoside is very stable and can penetrate, but it is not known whether it is converted to AA. Derivatives containing fat-soluble parts such as palmitate are designed for greater absorption but do not necessarily show better stability and it is not known whether these derivatives are efficiently converted in vivo. Recent studies suggest that encapsulation in a lipospheric form can promote transport into the lower layers of the epidermis and can ensure greater absorption. However, AA derivatives are not currently used in the field of skin cancers. Both its biodistribution through skin layers and through biological membranes has been little studied and the implications for its topical use are poorly understood. It has long been believed that this hydrophilic molecule only needs protein transporters to cross biological membrane barriers. The distribution of AA within the body requires bi-directional fluxes and, so far, only the AA transporters (SVCTs), facilitating its intake by cells have been identified. However, recent data suggest the implication of a possible passive transport of AA [18,19]. Experimental tests have shown that AA binds efficiently to the lipid bilayer interface and slowly crosses its hydrophobic core. It has been shown, using lipid membrane models, that AA crosses the lipid bilayer by passive diffusion and that the permeability coefficient depends on its protonation and high concentration gradients [19]. The permeability of the deprotonated AA (AscH^−^) has been determined to be orders of magnitude less than its protonated form (AscH_2_), so the AA must be protonated at the lipid bilayer interface before entering the hydrophobic region (Figure 4). It has been shown [18] that in AA homeostasis, the SVCT transporter is able to generate an AA concentration gradient across the plasma membrane by increasing its intracellular concentration. In turn, the concentration gradient generated by SVCT activity pushes the passive diffusion of AA out of the cell. These studies show that the AA must be protonated at the interface of the lipid bilayer, before entering the hydrophobic region. Therefore, to ensure its passive diffusion, the concentration and pH are decisive chemical-physical parameters. In light of all the above considerations, we examine the potential clinical translation of AA by focusing on skin cancers and in particular epitheliomas.

## 8. Skin Cancer and Incidence

Skin tumors represent on average 10–15% of all malignant tumors cancers. This heterogeneous but common group of primitive neoplasms consists of about 90–95% epitheliomas, 5% melanomas, and about 1% of the cases of rarest skin tumors. The epitheliomas, which originate from the keratinocytes of the epidermis, are represented in 75% and 15% respectively by the Basocellular and Squamocellular histotypes with a ratio between males and females of 2–3/1 [20,21,22,23]. These neoplasms, which apparently seem to have less oncological relevance, are characterized by considerable epidemiological, demographic and clinical-prognostic differences. In some geographical areas, their incidence rate sometimes exceeds 50% of that of other cancers, which is unequivocally related to latitude, race, and age. These tumors have a high incidence, mainly in the elderly population. In Australia, squamous cell carcinoma (SCC-321/100,000/’95) and basocellular carcinoma (BCC-788/100,000/’95) have the highest incidence, compared to other parts of the world. In some areas, their frequency has reached 2074/cases/100.000 inhabitants with an expense pairs to 232.2 million dollars per year [24]. In Italy in the period 1998–2002 the NMSC (75% BCC and 15–20% SCC), were respectively at one place in the male sex and two place in the female sex, after breast cancer, with a percentage of 15.2% and 14.8% of the total tumors, as reported by the Italian Cancer Registry Association. In Italy, an average of 119.4 cases of non-melanocytic skin cancer per 100,000 men and 90.7 per 100,000 women were diagnosed each year. The risk of having a lifetime diagnosis of NMSC skin cancer (between 0 and 74 years) is 6.47 among males (equal to one case per 16 men) and 4.08 among females (1 case per 24 women). The probability of developing a BCC over the lifetime varies from 11% to 28% and from 1.5% to 11% for the SCC [25].

## 9. Etiology and Treatment of NMSC

NMSCs occur mainly in exposed photo areas, with a frequency of 80% in the cephalic extremity. The complex anatomy of the face and scalp compared to other locations often makes surgery difficult. In these regions, the knowledge of topographical anatomy and physiology is vital for the planning of a surgical act that avoids severe aesthetic or functional defects. Sometimes, to achieve a complete resection, partial or total sacrifice of structures such as lips, eyelids, or tear ducts is required. In the cervical facial area, at a late diagnosis of extended forms of BCC mutilating and repeated recurrent treatments, while in cases of aggressive SCC, when these spread with distant metastasis may be impossible to perform effective treatment. Regarding the treatment of NMSC, certainly the complete surgical excision or RT, offers the highest and most reliable rates of disease control and a better prognosis.

The results of the exclusive radiant treatment are similar to those of surgery with a control rate in years of approximately 90%. Radiant adjuvant treatment after non radical surgery reduces the risk of local recurrence from 50% to 20–25% [26,27].

In the treatment of BCC, in addition to radiotherapy were described further conservative treatments although with higher rates of persistence/ recurrence, that are photodynamic therapy, cryotherapy, curettage, electrodissection, use of intralesional alpha interferon, and the topical use of imiquimod. In the primary BCC for non-surgical treatments percentages of persistence or recurrence are reported: from 0% to 21% for cryotherapy; from 3% to 42% for curettage and electro-desiccation; from 2% to 30% for intralesional alpha interferon; 0% to 3% for photodynamic therapy; from 7% to 16% for radiotherapy; from 2% to 10% for surgical excision; from 7% to 21% for topical use of 5-fluorouracil; from 0% to 31% for topical use of imiquimod. In the literature, many studies certified the utility of the topical use of imiquimod and 5-fluorouracil fort the treatment of NMSC.

The topical treatments reported for NMSC (mainly for BCC) include: topical chemical destruction with 5-fluorouracil and topical immunomodulatory therapy with Imiquimod.

SCCs are generally not treated topically: Imiquimod and 5-fluorouracil are not approved for the treatment of SCC and there are few data on their therapeutic efficacy.

Nevertheless, we found three studies regarding the use of AA for the treatment of NMSCs, including SCCs.

These three studies provide us with an overview for a potential new treatment and give us empirical confirmation of the mechanism of AA in the skin. In this context, AA shows a not yet described mechanism of action for NMSC skin cancers and new research in this area could be considered for the development of new drugs, based on a mechanism of action similar to AA.

AA-based treatment could be used in addition to established therapies or could represent an additional therapeutic option. Topical treatments used for these cancers are mainly encouraged due to the high incidence of NMSC, especially in the elderly population. The high costs associated with the management of this category of patients related to hospitalizations and surgery mean that there is interest in the search for simpler and cheaper treatments.

Furthermore, a simple topical treatment that can be performed before surgery, to favor a more conservative approach, would mean a great improvement in clinical practice. NMSCs mainly affect the head and neck area and can lead to major mutilations (complete nose-ear resection), therefore, reducing the size of the lesions would lead to less disfigurement and improve the patient’s condition.

Although NMSCs can be effectively treated with traditional treatments, there is great interest in new topical treatments related to the reasons discussed above.

## 10. L-Ascorbic Acid for Topical Use in NMSC

In this review, we show how some clinical evidence has shifted the interest on this molecule for topical use in NMSC. The clinical cases [9,10,11] seem to confirm the rationale presented by Levine et al. [5] and Qi Chen et al. [6,7], according to which, AA would act as a pro-drug and how its pharmacokinetic plays a central role. These studies take into account the treatment of skin disease, considering the limitations related to biodistribution previously described and providing an empirical approach for topical treatment of BCC and SCC of the skin. It is therefore of interest to analyze how the chemical-physical characteristics of this formulation, can find confirmation in its clinical use. In this context, some considerations can be made. A topical formulation, with adequate concentrations of AA and pH values, can be prepared to obtain the best characteristics to ensure transport into the layers of the skin. AA in topical administration can generate Asc • and H_2_O_2_ directly in extracellular tissue, close to tumor cells, without implications related to its pharmacokinetics.

Asc • and H_2_O_2_ can be formed by site specific activation, mediated by metal proteins present in the tissues, bypassing the inhibitory action of the blood. Furthermore, continuous exposure over time to peroxide, capable of determining cytotoxicity towards tumor cells, would be more easily achievable than systemic administration.

In the clinical case shown by Pernice et al. [11] the use of a highly concentrated AA solution has been described. The authors describe the treatment of an ear SCC by reporting a total clinical and histological response. The formulation had a concentration equal to or greater than the solubility limit >33 g/100 mL in water and a low pH, between 1.2 and 3.5. The frequency of administration was daily and the drug was applied for a minimum of 4 h to a maximum of 12 h each day. An excellent result was achieved with complete tumor remission within one month, confirmed by biopsy. Interestingly, histology revealed a lymphocyte infiltrate after treatment. The presence of focal epidermal hyperplasia with hyperkeratosis and small crusts is described in post-treatment biopsies [11]. The underlying superficial dermis was characterized by fibrosis associated with focal lymphocytic inflammatory infiltrate and no residual neoplasm was evident. Although the mechanism of the immune response is not completely understood, it is plausible that H_2_O_2_-mediated cytotoxicity is accompanied by an inflammatory process and the presence of an immune response. The presence of immune infiltrates in biopsies performed on treated patients has also been described by Holló et al. [9], where the efficacy of topical AA for the treatment of BCC was evaluated. In the latter, seven cases of BCC were treated with a saturation solution (=33 g/100 mL), using a local bandage placed for at least 12 h, for a total of 22 weeks. Over an 18-month follow-up period, resolution of five cases of BCC was achieved, confirmed by a post-therapy biopsy, and only one case relapsed. Both studies show that this treatment is generally well tolerated and free from serious side effects. As reported [9], some patients experienced only local skin irritation limited to the treatment site (erythema with itching and burning).

In addition to this clinical evidence, there are only few data in the literature testing the use of a highly concentrated formulation of AA on the skin. A 2001 paper [28] investigated the absorption and distribution of AA, showing the critical importance of the pH of the formulation for percutaneous absorption. Experimental tests have shown that AA had to be formulated, in aqueous solution, at a high concentration and at a pH below 3.5, to be effective for absorption. The tests carried out on the concentration parameter, showed that the maximal concentration for optimal percutaneous absorption was 20% and that the tissue levels were saturated after three daily applications [28]. Generally, for topical administration, increasing the concentration gradient increases the amount of drug that can be transferred per unit of time. For example, resistance to some drugs can be overcome by using higher drug concentrations (see corticosteroids). In the case of AA, there is an increase in the amount absorbed using high concentrations of formulation. Chemically, AA is an organic acid which in aqueous solution and at high concentrations is able to greatly influence the pH and the saturated solutions can reach a pH < 3. Pinnell et al. [28] have shown experimentally that a low pH is essential for its absorption and release through the stratum corneum barrier, increasing its transfer efficiency, when the pH is reduced to 2.0. The acid pH (<3.5) allows the AA (pKa 4.2) to maintain a non-ionized form, thus improving its absorption and distribution. Experimental evidence on the mechanism of passive diffusion of AA and how it can significantly contribute to its absorption through the skin offers further evidence for the use of AA in this field.

AA is able to passively distribute itself across membranes [18,19] and therefore its transcutaneous absorption is directly influenced by the pH of the environment and the pKa of the molecule. It is known that the ionization of a molecule influences its diffusion, because cell membranes have a greater permeability towards its non-ionized form than the less fat-soluble ionized one. Generally, an acid vehicle improves the absorption of molecules with acid characteristics in the undissociated state and the concentration gradients determined by ion trapping can theoretically be very large if the difference in pH between the compartments considered is large. The difference in pH between the compartments plays a role in the absorption of drugs and in the case of acid topical formulations, the difference with the pH of the skin could determine a further contribution for its passage through the stratum corneum.

Further experimental information was provided to understand how the characteristics of the formulation influence the percutaneous absorption of AA [28]. In tests to determine whether DHA was preferable to topical AA, no increases in AA levels were found in the skin, starting with the DHA-based formulation. AA is an unstable molecule that tends to oxidize rapidly in air and water, so the pH of the solution plays an additional role in keeping the percentage of DHA low. The low pH of the formulation shifts the balance of the AA towards the reduced form of the molecule by reducing the presence of DHA and increasing its absorption. A further point to consider is related to the generation of the radical Asc •. AA spontaneously oxidizes at physiological pH and in the presence of oxygen concentrations, producing Asc • and DHA. This process can be accelerated by light, heat, increasing pH, and the presence of contaminating free metals [29]. Therefore, activation of AA (as a pro-drug) should only occur in extracellular fluids and the formulation should not contain potentially interfering metal species to promote radical formation. The formulations used in the clinical cases considered were prepared extemporaneously [11] without excipients and with highly purified H_2_O. It can be hypothesized that the presence of potential metal catalysts can initiate the auto-oxidation of AA, favoring the formation of its radical before tissue activation.

It should be noted that to preserve the AA from oxidation of air and light and to avoid its rapid degradation, the clinicians prepared the formulations extemporaneously and administered them immediately.

The cases described here show the use of a formulation prepared in a clinical setting, however it is useful to make some considerations from the technical–pharmaceutical point of view, in the hypothesis of a more extensive use.

AA tends to oxidize easily in light and in the presence of oxygen, so its use is limited by these conditions.

Patches or transdermal devices for its administration could be considered, reducing the contact of the AA with light and air or using stabilizers/excipients able of slowing the degradation of the AA.

To this end, the addition of antioxidants could be considered to avoid/slow down the oxidation of AA in the formulation; however, further studies should be conducted to verify whether the presence of additional substances could interfere with the mechanism of action of AA.

Furthermore, lipophilic derivatives could also be considered, to ensure better absorption, or derivatives less susceptible to oxidation. However, it is not clear whether chemical modifications of the AA, can interfere with the formation of its radical. Further studies on biochemistry and optimization of formulations will be needed in the future to overcome problems related to its stability.

A further step will be to investigate how the skin condition may affect the outcome of the treatment. The skin is in fact a dynamic organ and its characteristics can vary due to various factors such as: damaged skin, keratinization, metabolic states, but also age and diseases.

The dynamism of the skin has also been addressed by recent evidence that has linked the circadian clock to changes in skin physiology such as pH and permeability [30,31]. By exploiting the daily variations of circadian cycles in the functions of the epidermal barrier, the transcutaneous absorption of locally applied drugs could be improved.

In light of the above, it remains to be analyzed whether physiological, pathological, and circadian changes can lead to changes in AA absorption, distribution, and mechanism of action.

Studies have suggested that AA could be used as a treatment for BCC and SCC of the skin. The use of a topical formulation of AA, in a context of increasing incidence of these diseases, could be part of current therapeutic strategies. In conclusion, further studies are needed to understand the potential role of this treatment. Although the cases discussed here are preliminary clinical studies, it is important to consider them as a first step in the development of new topical therapies for skin cancers diseases.

## Data Availability

Not applicable.

## Abbreviations

AA	L-ascorbic acid
DHA	Dehydroascorbic acid
Asc •	Ascorbil radical
NMSC	Non-melanoma skin cancer
BCC	Basal cell carcinoma
SCC	Squamous cell carcinoma
SVCTs	Sodium-dependent vitamin C transporters

## References

[1] Cameron E., Campbell A. (1974). The orthomolecular treatment of cancer. II. Clinical trial of high-dose ascorbic acid supplements in advanced human cancer. Chem. Biol. Interact..

[2] Cameron E., Pauling L. (1976). Supplemental ascorbate in the supportive treatment of cancer: Prolongation of survival times in terminal human cancer. Proc. Natl. Acad. Sci. USA.

[3] Cameron E., Pauling L. (1978). Supplemental ascorbate in the supportive treatment of cancer: Reevaluation of prolongation of survival times in terminal human cancer. Proc. Natl. Acad. Sci. USA.

[4] Vissers M.C.M., Das A.B. (2018). Potential Mechanisms of Action for Vitamin C in Cancer: Reviewing the Evidence. Front. Physiol..

[5] Levine M., Padayatty S.J., Espey M.G. (2011). Vitamin C: A Concentration-Function Approach Yields Pharmacology and Therapeutic Discoveries. American Society for Nutrition. Adv. Nutr..

[6] Chen Q., Espey M.G., Sun A.Y., Lee J.H., Krishna M.C., Shacter E., Choyke P.L., Pooput C., Kirk K.L., Buettner G.R. (2007). Ascorbate in pharmacologic concentrations selectively generates ascorbate radical and hydrogen peroxide in extracellular fluid in vivo. Proc. Natl. Acad. Sci. USA.

[7] Chen Q., Espey M.G., Krishna M.C., Mitchell J.B., Corpe C.P., Buettner G.R., Shacter E., Levine M. (2005). Pharmacologic ascorbic acid concentrations selectively kill cancer cells: Action as a pro-drug to deliver hydrogen peroxide to tissues. Proc. Natl. Acad. Sci. USA.

[8] Pullar J.M., Carr A.C., Vissers M. (2017). The Roles of Vitamin C in Skin Health. Nutrients.

[9] Holló P., Jókai H., Hársing J., Soós G., Kárpáti S., Németh K. (2016). Topically applied ascorbic acid solution for the treatment of basal cell carcinoma (BCC). J. Am. Acad. Dermatol..

[10] Bánvölgyi A., Lőrincz K., Kiss N., Avci P., Fésűs L., Szipőcs R., Krenács T., Gyöngyösi N., Wikonkál N., Kárpáti S. (2020). Efficiency of long-term high-dose intravenous ascorbic acid therapy in locally advanced basal cell carcinoma: A pilot study. Adv. Dermatol. Allergol..

[11] Pernice C., Murri D., Valli R., Crocetta F.M., Iori M., Asti M., Ghidini A., Capponi P.C. (2021). Complete response of cutaneous SCC to topical treatment with ascorbic acid solution: A case report. Clin. Case Rep..

[12] Doskey C.M., Buranasudja V., Wagner B.A., Wilkes J.G., Du J., Cullen J.J., Buettner G.R. (2016). Tumor cells have decreased ability to metabolize H_2_O_2_: Implications for pharmacological ascorbate in cancer therapy. Redox. Biol..

[13] Miyoshi N., Oubrahim H., Chock P.B., Stadtman E.R. (2006). Age-dependent cell death and the role of ATP in hydrogen peroxide-induced apoptosis and necrosis. Proc. Natl. Acad. Sci. USA.

[14] Timoshnikov V.A., Kobzeva T.V., Polyakov N.E., Kontoghiorghes G.J. (2020). Kontoghiorghes. Redox Interactions of Vitamin C and Iron: Inhibition of the Pro-Oxidant Activity by Deferiprone. Int. J. Mol. Sci..

[15] Forman H.J., Bernardo A., Davies K.J. (2016). What is the concentration of hydrogen peroxide in blood and plasma?. Arch. Biochem. Biophys..

[16] Bielski B.H., Richter H.W., Chan P.C. (1975). Some properties of the ascorbate free radical. Ann. N.Y. Acad. Sci..

[17] Lykkesfeldt J., Tveden-Nyborg P. (2019). The Pharmacokinetics of Vitamin C. Nutrients.

[18] Łukawski M., Dałek P., Witkiewicz W., Przybyło M., Langner M. (2020). Experimental evidence and physiological significance of the ascorbate passive diffusion through the lipid bilayer. Chem. Phys. Lipid.

[19] Hannesschlaeger C., Pohl P. (2018). Membrane Permeabilities of Ascorbic Acid and Ascorbate. Biomolecules.

[20] Armstrong B.K., Kricker A. (2001). The epidemiology of UV induced skin cancer. J. Photochem. Photobiol..

[21] Wu S., Han J., Li W.Q., Li T., Qureshi A.A. (2013). Basal-Cell Carcinoma Incidence and Associated Risk Factors in US Women and Men. Am. J. Epidemiol..

[22] Batra S., Kelley L. (2002). Predictors of extensive subclinical spread in non-melanoma skin cancer treated with Mosh micrographic surgery. Arch. Dermatol..

[23] Breuninger H., Black B., Rassner G. (1990). Microstaging of squamous cell carcinomas. Am. J. Clin. Pathol..

[24] NHRMC (2008). Clinical Practice Guidelines. Non-Melanoma Skin Cancer: Guidelines for Treatment and Management in Australia.

[25] Mazzeo F., Forestieri P. (2003). Trattato di Chirurgia Oncologica.

[26] Lovett R.D., Perez C.A., Shapiro S.J., Garcia D.M. (1990). External irradiation of epithelial skin cancer. Int. J. Radiat. Oncol. Biol. Phys..

[27] Griep C., Davelaar J., Scholten A.N., Chin A., Leer J.W.H. (1995). Electron beam therapy is not inferior to superficial x-ray therapy in the treatment of skin carcinoma. Int. J. Radiat. Oncol. Biol. Phys..

[28] Pinnell S.R., Yang H., Omar M., Riviere N.M., DeBuys H.V., Walker L.C., Wang Y., Levine M. (2001). Topical L-Ascorbic Acid: Percutaneous Absorption Studies. Dermatol. Surg..

[29] Buettner G.R. (1988). In the absence of catalytic metals ascorbate does not autoxidize at pH 7: Ascorbate as a test for catalytic metals. J. Biochem. Biophys. Methods.

[30] Plikus M.V., Van Spyk E.N., Pham K., Geyfman M., Kumar V., Takahashi J.S., Andersen B. (2015). The circadian clock in skin: Implications for adult stem cells, tissue regeneration, cancer, aging, and immunity. J. Biol. Rhythms..

[31] De Assis L.V.M., Moraes M.N., de Lauro Castrucci A.M. (2019). The molecular clock in the skin, its functionality, and how it is disrupted in cutaneous melanoma: A new pharmacological target?. Cell Mol. Life Sci..

